# Multifocal invasive ductal breast cancer with osteoclast-like giant cells: a case report

**DOI:** 10.1186/1752-1947-5-85

**Published:** 2011-02-27

**Authors:** Georg Richter, Christoph Uleer, Thomas Noesselt

**Affiliations:** 1 Institute of Pathology, 31785, Hameln, Germany; 2 Mammography Screening Unit Lower Saxony South, D-31134 Hildesheim-Hameln-Göttingen, Germany; 3 Department of Gynecology, District Hospital Hameln, D-31785 Hameln, Germany

## Abstract

**Introduction:**

To the best of our knowledge, this is the first case report of a multifocal (trifocal) invasive carcinoma of the breast containing osteoclast-like giant cells.

**Case presentation:**

A 64-year-old Caucasian woman presented for routine mammography screening with three radiodense lesions in the lower inner quadrant of the right breast, a primary breast cancer. Microscopic examination showed three foci of invasive ductal carcinoma with multinucleated osteoclast-like giant cells. Osteoclast-like giant cells in breast cancer are a rare phenomenon. They are described in less than two percent of all breast cancers and occur in association with invasive ductal cancer and invasive lobular cancer. In addition, osteoclast-like giant cells have been described in several sarcomas and metaplastic carcinomas of the breast.

**Conclusion:**

To the best of our knowledge, this is the first report of a multifocal infiltrating ductal carcinoma of the breast containing osteoclast-like giant cells. This could be an indication for a possible early event in carcinogenesis associated with a biological event or secretion that indicates the differentiation and/or migration of stromal cells or macrophages.

## Introduction

Carcinoma of the breast containing osteoclast-like giant cells is uncommon and described in less than 2% of breast cancer patients [1-3]. In addition, osteoclast-like giant cells are described in a ductal carcinoma *in situ *and metaplastic carcinomas of the breast [4,5], although the stromal origin of the giant cells is unknown. Immunohistochemical and ultrastructural studies suggest that the osteoclast-like giant cells are of stromal histiocytic origin or might be differentiated from macrophages [6-9]. The characteristic multinucleated giant cells are found at the periphery of the tumor cells and within the glandular luminal spaces in primary in situ, invasive breast cancers and in metastases. We report the first case of a multifocal invasive ductal breast cancer with osteoclast-like giant cells.

## Case presentation

A 64-year-old Caucasian woman presented for routine mammography screening within the National Mammography Screening Program. She had no known family history of breast cancer and denied recent signs or symptoms of breast disease on her intake questionnaire.

**Figure 1:**
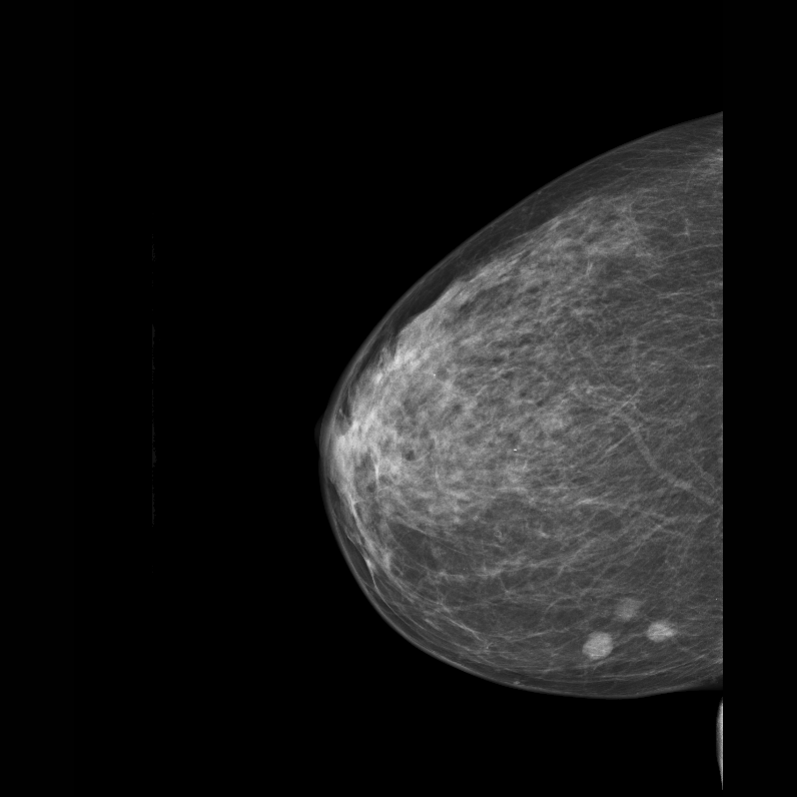
**Digital mammography (mediolateral projection)**.

The digital mammogram showed three radiopaque lesions in the lower inner quadrant of the right breast, which were readily detectable in both the mediolateral oblique and craniocaudal projection views (Figure 1). The density of the breast tissue was estimated as type 2 according to the classification system of the American College of Radiology (low-density, fibroglandular tissue). Round microcalcifications were found to be diffusely distributed in both breasts.

**Figure 2:**
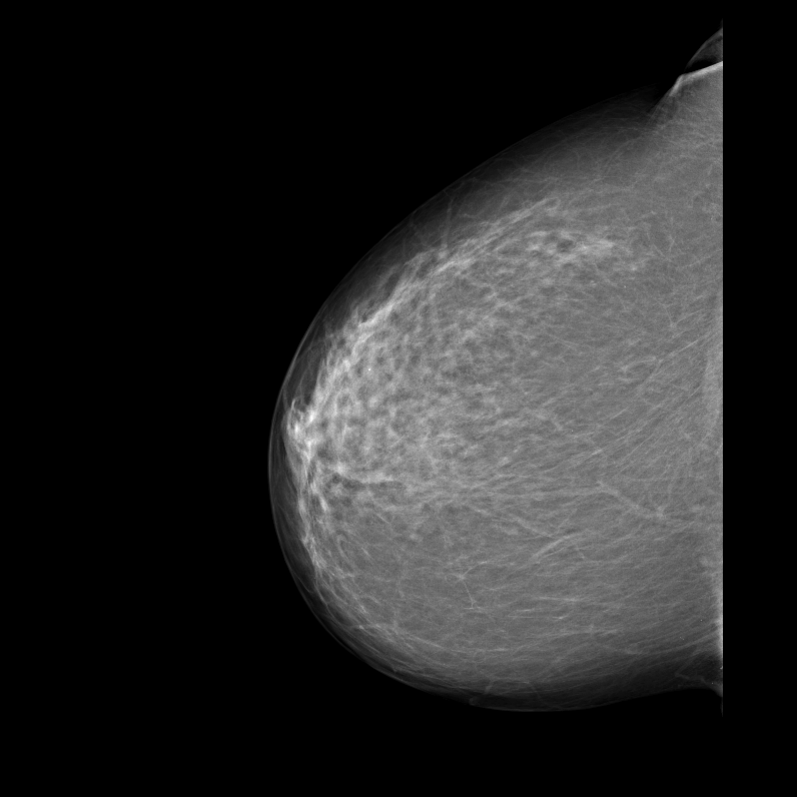
**Screening mammogram performed two years earlier than 2009**.

**Figure 3:**
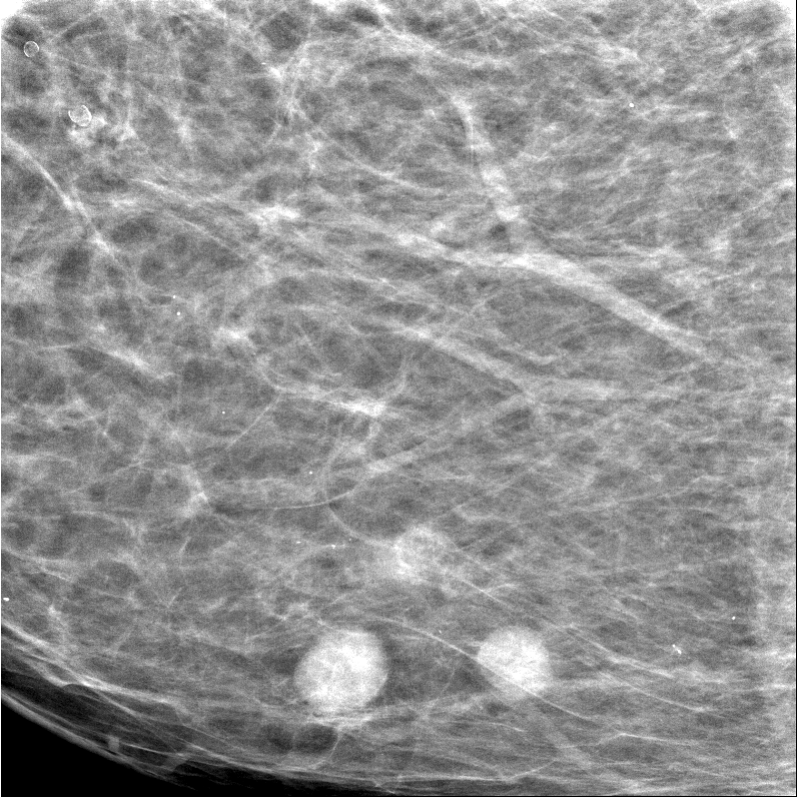
**Craniocaudal spot compression in digital mammogram view focused on the three lesions**.

Each of the three lesions in the right lower inner quadrant had slightly irregular margins and measured 0.7 cm × 0.9 cm. Since these lesions were absent in the previous screening mammogram performed two years earlier (Figure 2), they were considered suspicious for multifocal breast cancer (Breast Imaging Reporting and Data System (BI-RADS) category 4B). Therefore, the woman was called back into the screening center for further evaluation. A craniocaudal spot compression view focused on the three lesions was obtained. On this view, the radiodense lesions with irregular margins were easily distinguished from the surrounding fat tissue (Figure 3).

A breast ultrasound was performed, and in the right inner lower quadrant the lesions were visible as complex masses with irregular margins and inhomogeneous internal echoes (BI-RADS analogue 4). The left breast as well as the ipsilateral and contralateral axillary lymph nodes were normal.

Since there was a good correlation between the suspicious mammographic lesions and the ultrasound image, an ultrasound-guided core needle biopsy was performed for each of the three tumors. Five specimens were thereby obtained confirming the diagnosis of multifocal invasive cancer. Because of the multifocal character of the breast cancer, a bilateral breast magnetic resonance imaging (MRI) scan was obtained to exclude further lesions. Eleven days after the woman's first contact with the screening center, the interdisciplinary tumor board recommended breast-conserving surgery and sentinel node biopsy following preoperative needle localization of the tumor.

As the foci were lying close together in one quadrant, a breast-preserving operation could be performed. Additionally, a sentinel node marking and a sentinel node biopsy were induced by clinically and sonographically negative axillary results.

For the operation, the three foci were portrayed preoperatively using sonography with a needle marking. First, the sentinel node biopsy was carried out. After marking with Nanocoll technetium-99 m (Gipharma Sri, Saluggia Vercelli, Italy) a sentinel node was portrayed in the right axilla by lymphscintigraphy. Intraoperative 1.5 ml Acid Blue solution (Guerbet, Sulzbach, Taunus, Germany) was additionally injected peritumorally, and the axilla was examined using a gamma probe. A focus of heightened activity showed up in the right lower axilla. A radioactively marked lymph node was found during the preparation of the axilla. There were no other foci of heightened activity. The frozen section examination of the sentinel node was negative.

Afterward a breast-preserving excision including a skin spindle was performed. The excision contained all the invasive foci and presented clear margins. For reconstruction, intramammary wound closure with an advancement plastic of breast tissue was installed into the defect. Postoperative proper wound healing was observed.

**Figure 4:**
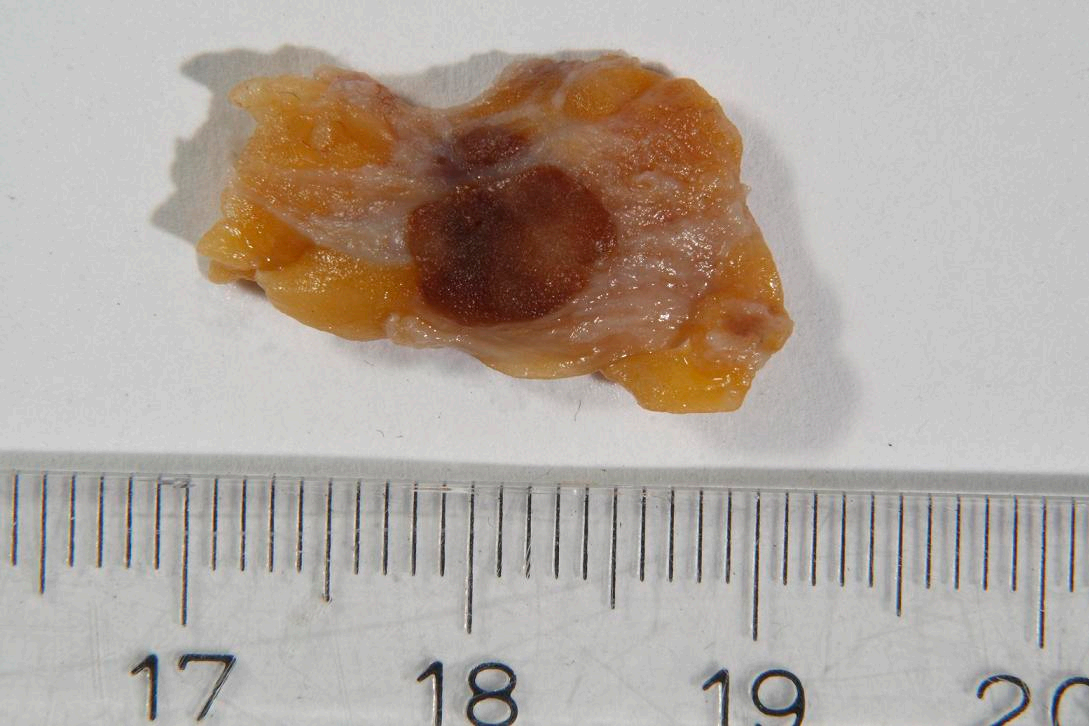
**Macrophotography of the greatest focus**.

An intraoperative investigation of a breast specimen weighing 31 g and measuring 8 cm × 5 cm × 3 cm was undertaken to examine the resection margins. Also, one sentinel node was examined to exclude metastases. In the macroscopic examination, three neighboring foci showing a brown incision surface and measuring 1.2 cm, 0.8 cm and 0.6 cm were found (Figure 4). The specimens were routinely fixated in 4% buffered formalin, embedded in paraffin and sectioned into 3 μm to 4 μm thick sections. Then the specimens were routinely stained with hematoxylin and eosin. Also, they were immunohistochemically stained with the primary antibodies Cytokeratin 5/6 (Cell Marque) (Roche Ventana Medical Systems, Illkirch, France), Cytokeratin 7 (Roche Ventana), Vimentin (Roche Ventana), CD68 (Roche Ventana), Estrogen Receptor (Roche Ventana), Progesterone Receptor (Roche Ventana), human epidermal growth factor receptor 2 (HER2) (Roche Ventana) and Ki-67 antigen (Roche Ventana) using the ultraView™ Universal Alkaline Phosphatase Red Detection Kit (Roche Ventana) on the Roche Ventana benchmark with on-slide positive controls. All Ventana kits are ready to use.

**Figure 5:**
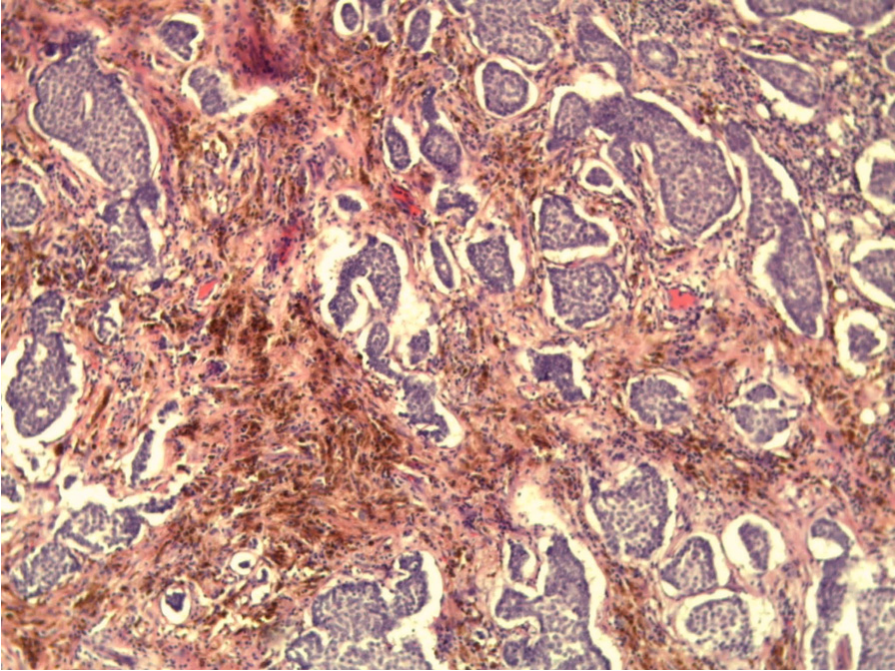
**Microphotography (hematoxylin and eosin staining; original magnification, × 200)**.

**Figure 6:**
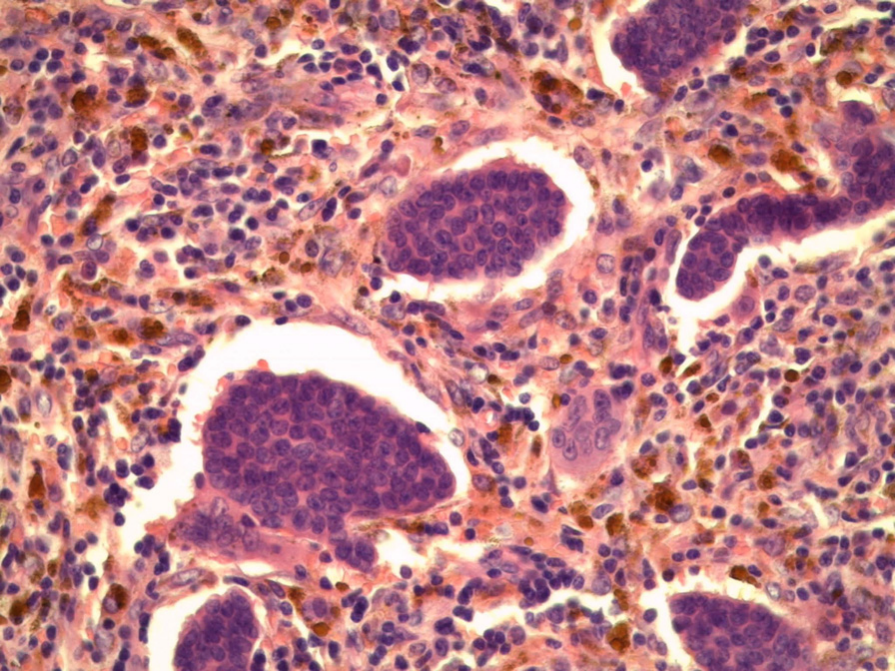
**Microphotography (hematoxylin and eosin staining; original magnification, × 400)**.

**Figure 7:**
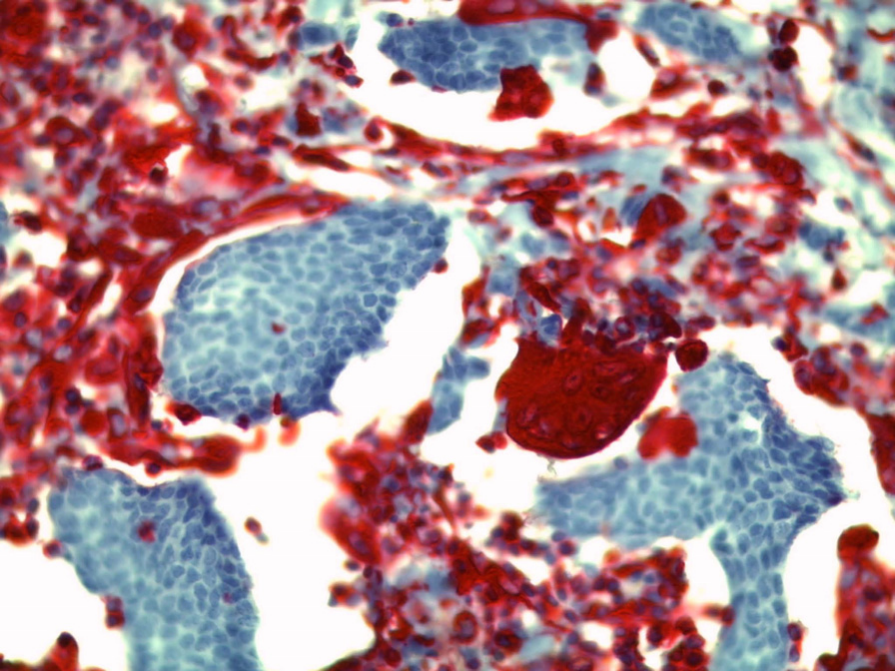
**Immunohistochemical positive reaction of the stromal cells for vimentin (original magnification, × 400)**.

**Figure 8:**
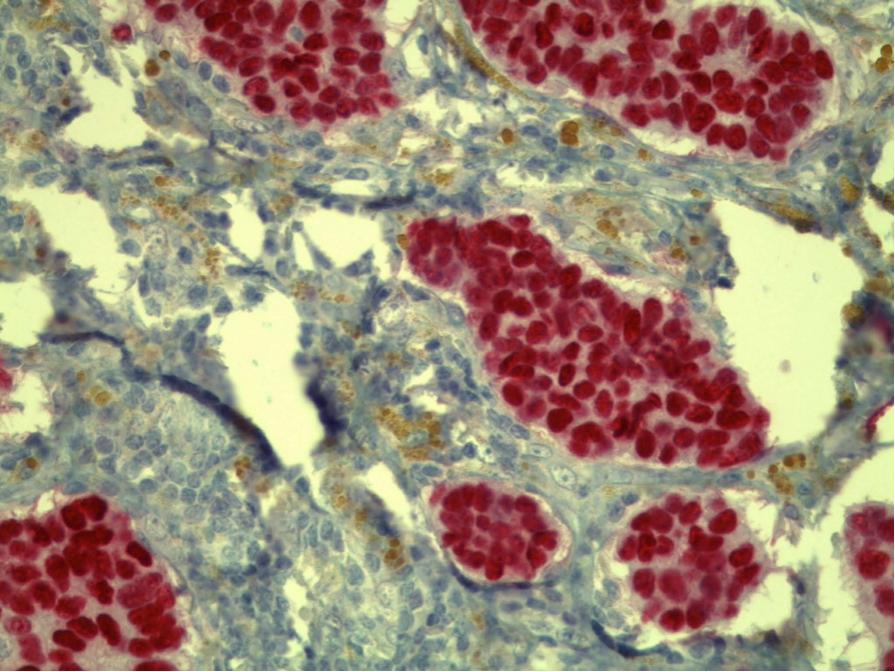
**Immunohistochemical positive reaction with antibody against the estrogen receptor in the tumor cells (original magnification, × 400)**.

**Figure 9:**
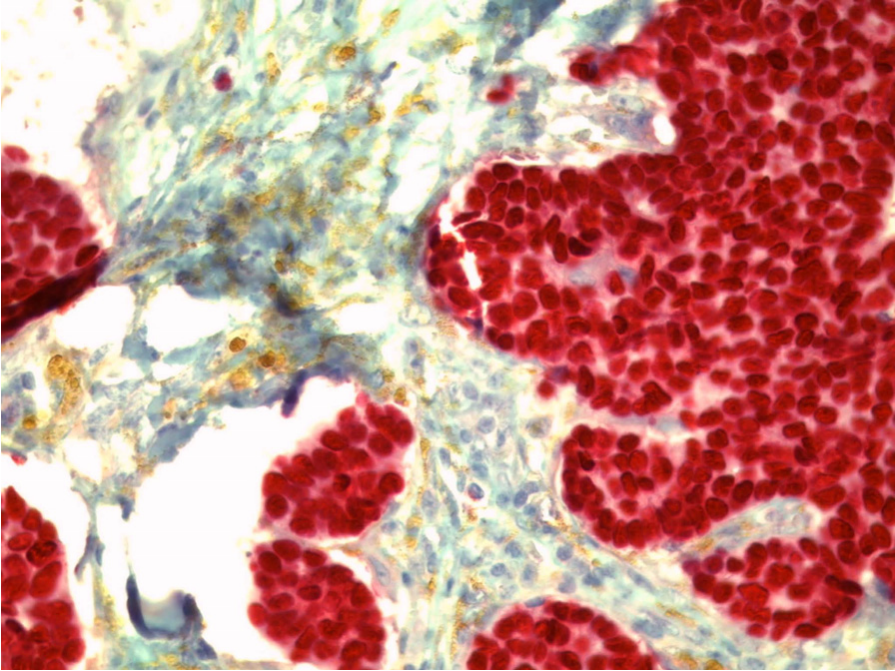
**Immunohistochemical positive reaction with antibody against the progesterone receptor in the tumor cells (original magnification, × 400)**.

**Figure 10:**
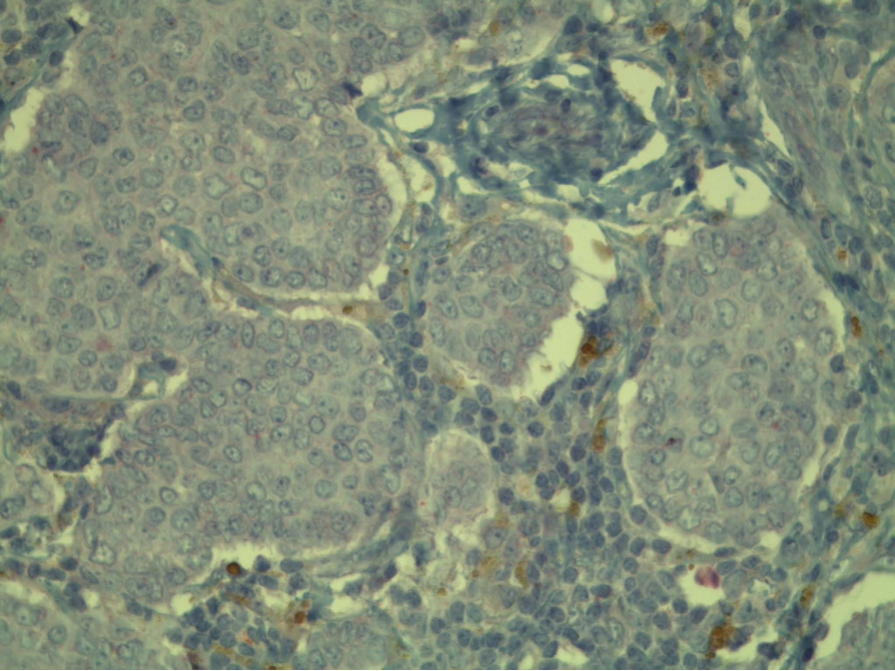
**Immunohistochemical negative reaction with antibody against human epidermal growth factor receptor 2(HER2)/neu in the tumor cells (original magnification, × 400)**.

**Figure 11:**
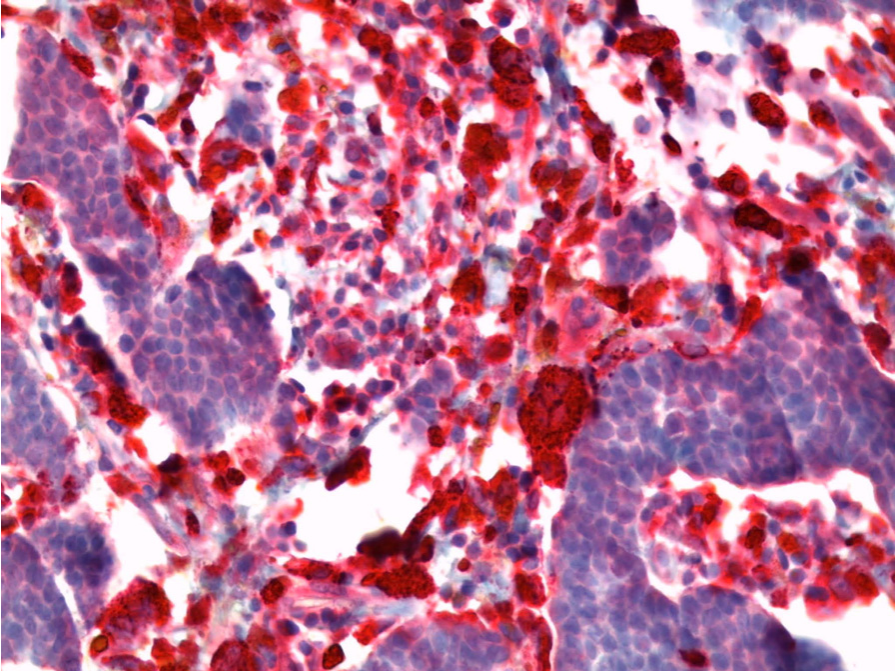
**Immunohistochemical positive reaction with antibody against CD68 in the giant cells (original magnification, × 400)**.

Microscopic examination showed three foci of an invasive ductal carcinoma with a moderate amount tubule formations, moderate nuclear pleomorphism with visible nucleoli and 8 mitoses/10 high-power fields following the grading of Elston and Ellis [12] (Figures 5 and 6). No squamous cells or other metaplasia were exhibited in any of the foci. On the basis of immunohistochemistry, we detected a positive reaction for cytokeratin 7 and a negative reaction for cytokeratin 5/6 and vimentin in the epithelial tumor cells (Figure 7). Using the Allred score, the estrogen and progesterone receptors were similarly positive in all three foci (Proportion Score 5 + Intensity Score 3 = Total Score 8) (Figures 8 and 9), and in accordance with the Dako score, we detected a HER2 score of 0 (negative) (Figure 10). The Nottingham grade for invasive cancer was 0.2 × 1.2 + (G) 2 + 0 = 3.4; Nottingham Prognostic Index score 3.4 (intermediate). The giant cells contained numerous uniform nuclei and eosinophilic cytoplasm and had an appearance identical to an osteoclast. Immunohistochemically, the giant cells showed a positive reaction for vimentin and CD68 (Figure 11) and a negative reaction for the cytokeratins and the hormone receptors.

Owing to the tumor entity, there is a heightened risk of a systemic recurrence. Anthracycline-based chemotherapy with four cycles of epirubicin 90 mg/m^2 ^and cyclophosphamide 600 mg/m^2 ^was added as there was an overexpression of plasminogen activator inhibitor-1 (PAI-1, 54 ng/mg; urokinase plasminogen activator, 1.1 ng/mg) as a prediction of the effectiveness for adjuvant chemotherapy. Our patient was treated with continuation of adjuvant therapy with aromatase inhibitor and radiation of the breast with 50.4 dye plus local boost radiotherapy of the tumor bed.

## Conclusion

To the best of our knowledge, we present the first case report of a multifocal invasive ductal breast cancer with osteoclast-like giant cells. Osteoclast-like giant cells are rare in breast cancer, and the prognostic significance of their presence is uncertain [10,11]. Immunohistochemical and ultrastructural studies suggest that the osteoclast-like giant cells are of stromal histiocytic origin or possibly are terminally differentiated from macrophages. We detected three neighboring foci of an invasive ductal breast cancer with giant cells containing numerous uniform nuclei and eosinophilic cytoplasm adjacent to the epithelial tumor cells, an appearance identical to osteoclasts. This could be an indication for a possible early event in carcinogenesis associated with a biological event or secretion that indicates the differentiation and/or migration of stromal cells or macrophages.

## Competing interests

The authors declare that they have no competing interests.

## Consent

Written informed consent was obtained from the patient for publication of this case report and accompanying images. A copy of the written consent is available for review by the Editor-in-Chief of this journal.

## Authors' contributions

UC analyzed and interpreted the mammography and ultrasound. TN performed the operation and administered chemotherapy. GR performed the histological examination and was a major contributor in writing the manuscript. All authors read and approved the final manuscript.

## References

[B1] Rosen PP Mammary carcinoma with osteoclast-like giant cells Rosen's Breast Pathology 2001 Philadelphia: Lippincott Williams & Wilkins 517 526

[B2] Holland R van Haelst UJ Mammary carcinoma with osteoclast-like giant cells: additional observations on six cases Cancer 1984 53 1963 1973 10.1002/1097-0142(19840501)53:9<1963::AID-CNCR2820530927>3.0.CO;2-N 6704923

[B3] Cai N Koizumi J Vazquez M Mammary carcinoma with osteoclast-like giant cells: a study of four cases and a review of literature Diagn Cytopathol 2005 33 246 251 10.1002/dc.20341 16138376

[B4] Krishnan C Longacre T Ductal carcinoma in situ of the breast with osteoclast-like giant cells Hum Pathol 2006 37 369 372 10.1016/j.humpath.2005.11.012 16613333

[B5] Wargotz ES Norris HJ Metaplastic carcinomas of the breast: V. Metaplastic carcinoma with osteoclastic giant cells Hum Pathol 1990 21 1142 1150 10.1016/0046-8177(90)90151-T 2227922

[B6] Pettinato G Petrella G Manco A di Pisco B Salvatore G Angrisani P Carcinoma of the breast with osteoclast-like giant cells: fine needle aspiration cytology, histology and electron microscopy of 5 cases Appl Pathol 1984 2 168 178 6544601

[B7] Sano M Kikuchi K Zhao C Kobayashi M Nakanishi Y Nemoto N Osteoclastogenesis in human breast carcinoma Virchows Arch 2004 444 470 472 10.1007/s00428-004-0989-1 15014987

[B8] Athanasou NA Wells CA Quinn J Ferguson DP Heryet A McGee JO The origin and nature of stromal osteoclast-like multinucleated giant cells in breast carcinoma: implications for tumour osteolysis and macrophage biology Br J Cancer 1989 59 491 498 10.1038/bjc.1989.102 2713238 PMC2247156

[B9] Shishido-Hara Y Kurata A Fujiwara M Itoh H Imoto S Kamma H Two cases of breast carcinoma with osteoclastic giant cells: are the osteoclastic giant cells pro-tumoural differentiation of macrophages? Diagn Pathol 2010 5 55 10.1186/1746-1596-5-55 20731838 PMC2936386

[B10] Agnantis NT Rosen PP Mammary carcinoma with osteoclast-like giant cells: a study of eight cases with follow up Am J Clin Pathol 1979 72 383 389 474518 10.1093/ajcp/72.3.383

[B11] Saimura M Fukutomi T Tsuda H Tanaka SA Nanasawa T Breast carcinoma with osteoclast-like giant cells: a case report and review of the Japanese literature Breast Cancer 1999 6 121 126 10.1007/BF02966918 11091703

[B12] Elston CW Ellis IO Pathological prognostic, factors in breast cancer. 1. The value of histological grade in breast cancer: experience from a large study with long term follow up Histopathology 1991 19 403 410 10.1111/j.1365-2559.1991.tb00229.x 1757079

